# Seroepidemiology of SARS-CoV-2 on a partially vaccinated island in Brazil: Determinants of infection and vaccine response

**DOI:** 10.3389/fpubh.2022.1017337

**Published:** 2022-11-14

**Authors:** José Cerbino-Neto, Igor Tona Peres, Margareth Catoia Varela, Luciana Gomes Pedro Brandão, Juliana Arruda de Matos, Luiz Felipe Pinto, Marcellus Dias da Costa, Márcio Henrique de Oliveira Garcia, Daniel Soranz, Maria de Lourdes de Sousa Maia, Marco Aurélio Krieger, Rivaldo Venâncio da Cunha, Luiz Antonio Bastos Camacho, Otavio Ranzani, Silvio Hamacher, Fernando Augusto Bozza, Gerson Oliveira Penna

**Affiliations:** ^1^Municipal Health Department of Rio de Janeiro, Rio de Janeiro City Government, Rio de Janeiro, Brazil; ^2^National Institute of Infectious Disease Evandro Chagas, Oswaldo Cruz Foundation (FIOCRUZ), Rio de Janeiro, Brazil; ^3^D'Or Institute for Research and Education, Rio de Janeiro, Brazil; ^4^Department of Industrial Engineering and Tecgraf Institute, Pontifical Catholic University of Rio de Janeiro, Rio de Janeiro, Brazil; ^5^National Institute of Traumatology and Orthopedics Jamil Haddad, Rio de Janeiro, Brazil; ^6^School of Medicine, Federal University of Rio de Janeiro, Rio de Janeiro, Brazil; ^7^National School of Public Health, Oswaldo Cruz Foundation (FIOCRUZ), Rio de Janeiro, Brazil; ^8^Biomanguinhos Institute, Oswaldo Cruz Foundation (FIOCRUZ), Rio de Janeiro, Brazil; ^9^Vice Presidency of Production and Innovation in Health (VPPIS), Oswaldo Cruz Foundation (FIOCRUZ), Rio de Janeiro, Brazil; ^10^Coordination of Health Surveillance and Reference Laboratories, Oswaldo Cruz Foundation (FIOCRUZ), Rio de Janeiro, Brazil; ^11^Barcelona Institute for Global Health (ISGlobal), CIBER Epidemiología y Salud Pública, Universitat Pompeu Fabra, Barcelona, Spain; ^12^Pulmonary Division, Hospital das Clínicas (HCFMUSP), Heart Institute (InCor), Universidade de Sáo Paulo, Sáo Paulo, Brazil; ^13^Tropical Medicine Centre - University of Brasília and Fiocruz School of Government, Brasília, Brazil

**Keywords:** seroepidemiologic studies, COVID-19, antibody response, seropositivity, risk factors, vaccine

## Abstract

**Background:**

A vaccination campaign targeted adults in response to the pandemic in the City of Rio de Janeiro.

**Objective:**

We aimed to evaluate the seroprevalence of SARS-CoV-2 antibodies and identify factors associated with seropositivity on vaccinated and unvaccinated residents.

**Methods:**

We performed a seroepidemiologic survey in all residents of Paquetá Island, a neighborhood of Rio de Janeiro city, during the COVID-19 vaccine roll-out. Serological tests were performed from June 16 to June 19, 2021, and adjusted seropositivity rates were estimated by age and epidemiological variables. Logistic regression models were used to estimate adjusted ORs for risk factors to SARS-CoV-2 seropositivity in non-vaccinated individuals, and potential determinants of the magnitude of antibody responses in the seropositive population.

**Results:**

We included in the study 3,016 residents of Paquetá (83.5% of the island population). The crude seroprevalence of COVID-19 antibodies in our sample was 53.6% (95% CI = 51.0, 56.3). The risk factors for SARS-CoV-2 seropositivity in non-vaccinated individuals were history of confirmed previous COVID-19 infection (OR = 4.74; 95% CI = 3.3, 7.0), being a household contact of a case (OR = 1.93; 95% CI = 1.5, 2.6) and in-person learning (OR = 2.01; 95% CI = 1.4, 3.0). Potential determinants of the magnitude of antibody responses among the seropositive were hybrid immunity, the type of vaccine received, and time since the last vaccine dose. Being vaccinated with Pfizer or AstraZeneca (Beta = 2.2; 95% CI = 1.8, 2.6) determined higher antibody titers than those observed with CoronaVac (Beta = 1.2; 95% CI = 0.9, 1.5).

**Conclusions:**

Our study highlights the impact of vaccination on COVID-19 collective immunity even in a highly affected population, showing the difference in antibody titers achieved with different vaccines and how they wane with time, reinforcing how these factors should be considered when estimating effectiveness of a vaccination program at any given time. We also found that hybrid immunity was superior to both infection-induced and vaccine-induced immunity alone, and online learning protected students from COVID-19 exposure.

## Introduction

From the beginning of the COVID-19 pandemic until May 2022, Brazil has registered over 30,502,501 cases and 663,816 deaths, the third country with more cases and the second in the number of deaths (1). Despite the existence of information systems recording SARS-CoV-2 infections, diagnostic tests for COVID-19, and the number of vaccine doses administered, it is difficult to predict the range and degree of immunity at the populational-level. The high incidence of asymptomatic and oligosymptomatic infections, combined with the irregularity in the availability of confirmatory tests and the weaknesses of information systems, add uncertainties to the data on the cumulative incidence of COVID-19 in the population. On the other hand, a vaccination program with four different vaccines, each with different efficacy and immunogenicity, makes it difficult to estimate the level of collective protection in the vaccinated population. Regional and local differences in implementing non-pharmacological interventions such as social distancing and masks mandates add another layer of complexity to this analysis in Brazil and in many countries.

Seroepidemiological surveys provide a picture of collective immunity in a given area, an essential tool for situation diagnosis and health intervention planning. Since the pandemic's beginning, various forms of serological surveys have been used to assess the burden of COVID-19 and plan new control actions. The COVID-19 vaccination campaign in Rio de Janeiro city, Brazil, started on January 2021, prioritizing high-risk groups as healthcare workers and residents of long-term care facilities, and followed an age-based calendar beginning with the elderly (85 years or more). By June 2021, vaccine had been offered to all high-risk groups and to people 60 years or more, and a pilot mass vaccination intervention aiming at all adults and adolescents living in one neighborhood of Rio de Janeiro was proposed. We conducted a seroepidemiologic survey in the chosen area, the Paquetá Island neighborhood, before this mass immunization intervention to determine seroprevalence of SARS-CoV-2 antibodies and factors associated with seropositivity.

## Materials and methods

### Study design and participants

Rio de Janeiro is the second-largest city in Brazil, with an estimated population of 6.77 million habitants (2). Paquetá Island is a neighborhood of Rio de Janeiro city, with an area of 1,216 km^2^ and 3,612 residents, according to the 2022 census performed in Paquetá (3). Motorized vehicles are not allowed on the Island, which is accessed only by sea. The main transportation to Paquetá is done by a regular ferry, a 1-h ride from downtown Rio de Janeiro. With a Human Development Index of 0.822 (Rio de Janeiro city 0.842), the Island is a popular tourist destination, famous for its beaches and historical sites.

Considering its demographic, geographic, and administrative characteristics, Paquetá was chosen for a mass vaccination pilot study in June 2021, accelerating the vaccine rollout to all adults and adolescents on the Island, while at that point, in the rest of Brazil, only the elderly and healthcare workers were eligible for COVID-19 immunization. Before the mass vaccination started, we conducted a cross-sectional seroepidemiological survey on the Island from June 16 to 19, 2021, to understand the baseline epidemiological scenario.

Background information on COVID-19 notified cases, genomic surveillance and vaccine doses for Rio de Janeiro city and Paquetá Island from the beginning of the pandemic (March 2020) to the date of the survey (June 2021) was obtained from official health information systems (E-SUS, SIVEP, SI-PNI) (4–6). Cumulative incidence rates and vaccination coverage were calculated using population data from the national bureau of geography and statistics (IBGE) (2, 3). The study was approved by the National Research Ethics Committee (CONEP).

### Seroepidemiological survey

All residents of Paquetá Island were eligible for the study. Adult participants or legal guardians of minors signed the informed consent form (ICF), and answered a questionnaire to collect demographic, socioeconomic, mobility and epidemiologic data, including risk of COVID-19 exposure. Informed vaccination status was ascertained using the SI-PNI (national immunization program information system).

Serological tests were performed for all participants using two different methods. Adults collected 8 mL blood samples to establish a biobank and to perform a quantitative SARS-CoV-2 Anti-S IgG (Architect II, Abbott—Chicago, Illinois, EUA). This test is an automated chemiluminescent microparticle immunoassay (CMIA) which has shown a good correlation with neutralizing antibodies and has a reported sensitivity of 99.4% and specificity of 99.2% (7, 8). For children and adolescents (ages 0–18 years old), a whole-blood fingerpick was obtained to perform a point-of-care (POC) lateral-flow rapid test (Fastep, Azure Tech. Co., Ltd.—Hangzhou, Zhejiang, China) for anti-N and anti-S1 IgG and IgM detection (9), with results double-checked by two different study professionals. This rapid test has a reported sensitivity of 100% and specificity of 98.8% (10), and has shown equivalent results when compared to other serological methods (11). All serological tests followed the manufacturer use recommendations.

### Outcomes

Primary outcome was the presence of antibodies against SARS-CoV-2 (analyzed as a binary outcome). Secondary outcome was the antibody titers (continuous outcome restricted to the individuals who were seropositive).

### Statistical analysis

To calculate the seroprevalence of COVID-19 antibodies in Paquetá Island, we included all participants with a conclusive IgG test result in the analysis. We also calculated the seroprevalence by four age groups, children (ages 0–11), adolescents (ages 12–17), adults (ages 18–59) and elderly (ages 60+).

To describe the seroprevalence by demographic, epidemiological and clinical variables, we excluded participants with vaccination inconsistencies (duplicity of doses; more than one on the same day; second doses without first dose) and those with missing variables information. We evaluated different subgroups of the study population according to the purpose of the analysis. First, to examine the association of epidemiological factors and infection-induced immunity, we used an unvaccinated group, excluding all participants who had received any COVID-19 vaccine before the survey. Then, on the immunogenicity analysis, we excluded the participants tested with the POC qualitative test and included only the adults tested with the quantitative CMIA serology. For the binary categorical outcome, we used this whole group and, for the continuous outcome of log-transformed (base 10) antibody titers, we restricted the analysis to seropositive participants. We adjusted the crude seropositivity rates based on the serology tests estimates of sensitivity and specificity (12).

Logistic regression models were used to estimate adjusted ORs, 95% (CIs) and associated pairwise *p*-values for risk factors to SARS-CoV-2 seropositivity in non-vaccinated individuals, and potential determinants of antibody responses in the seropositive population. We selected for multivariate logistic regression the features that were statistically significant in the univariate regression, removing those with high collinearity with other features. We used boxplots (median, IQR) and other plots to evaluate the log10 antibody titers across vaccines and over the time. We considered a significance level of 5% for all statistical analyses.

All analyses were conducted in R statistical software version 4.0.3.

## Results

Paquetá Island COVID-19 cumulative incidence from March 2020 to June 2021 was 797.3 cases/10,000 hab, compared to 576.5 cases/10,000 hab for the city of Rio de Janeiro (Supplementary Figure 1). Predominant circulating SARS-CoV-2 variants during this period were B.1, B.1.1.33, B.1.1.28, Gamma and Zeta. The first isolation of the Delta variant was on late June 2021, after the conclusion of the seroepidemiological survey (Supplementary Figure 2). Vaccination coverage with at least one dose of a COVID-19 vaccine was 46.1% in Paquetá and 38.4% in Rio de Janeiro city at the start of the study (Supplementary Figure 1).

### Seropositivity rates

From June 16 to June 19, 2021, 3,016 residents of Paquetá were included in the study, 83.5% of the island population. The crude seroprevalence of COVID-19 antibodies in our sample was 53.6% (95% CI: 51.0–56.3). The seropositivity by age group was 24.9% (95% CI: 19.9–30.8) for children (ages 0–11), 18.1% (95% CI: 13.8–23.3) for adolescents (ages 12–17), 50.3% (95% CI: 47.0–53.9) for adults (ages 18–59) and 93.8% (95% CI: 86.6–101.5) for the elderly (ages 60+).

To describe the serological status by each epidemiological variable and for the regression models, we excluded 56 individuals with inconsistent or incomplete information and 41 participants with unascertained vaccination status (flowchart of study inclusion in Supplementary Figure 3). Therefore, 2,919 residents remained for this analysis. The sample is well-distributed among age groups, with seropositivity rates increasing with age (Table 1 for vaccinated and Table 2 for unvaccinated individuals). Among the participants, 1,832 (63.0%) were unvaccinated, with 31% (CI: 28.5, 33.7) of them testing positive for SARS-CoV-2 IgG. Seropositivity of unvaccinated children, adolescents and adults was 25, 18, and 35%, respectively. In contrast, in the 1,087 (37%) vaccinated residents seropositivity was 90.5% (95% CI: 85.0, 96.4). We considered vaccinated those who had received any COVID-19 vaccine at least 7 days prior to the survey, and we further stratified them in groups by type of vaccine and number of doses received. We found 571 participants vaccinated with the AstraZeneca vaccine, 109 of them with already two doses (97% seropositive) and 462 with only the first dose (83% seropositivity). 460 participants had received two doses of the CoronaVac vaccine (96% seropositive), and 56 were vaccinated with Pfizer, all of them with only the first dose as this vaccine had been recently introduced in the campaign and the interval for the second dose had not been met (95% seropositive).

**Table 1:** Demographic characteristics of the vaccinated population (*n* = 1,087).

		**SARS-CoV-IgG**			
**Variable**	**Total (%)**	**Negative *n*** **(%)**	**Positive *n*** **(%)**	**Adjusted** **Seropositivity** **(%)**	*P*-value*
**Age group (years)**					< 0.001
18–59	447 (41%)	62 (14%)	385 (86%)	86.1%	
60+	640 (59%)	41 (6.4%)	599 (94%)	93.6%	
**Sex**					0.68
Female	687 (63%)	67 (9.8%)	620 (90%)	90.2%	
Male	400 (37%)	36 (9.0%)	364 (91%)	91.0%	
**Arterial hypertension**					0.39
No	601 (55%)	61 (10%)	540 (90%)	89.9%	
Yes	486 (45%)	42 (8.6%)	444 (91%)	91.4%	
**Diabetes mellitus**					0.71
No	904 (83%)	87 (9.6%)	817 (90%)	90.4%	
Yes	183 (17%)	16 (8.7%)	167 (91%)	91.3%	
**Other comorbidities**					0.27
No	817 (75%)	82 (10%)	735 (90%)	90.0%	
Yes	270 (25%)	21 (7.8%)	249 (92%)	92.2%	
**Frequency of leaving the island**					0.37
Does not usually go out	210 (19%)	22 (10%)	188 (90%)	89.5%	
1–2 times a month	397 (37%)	31 (7.8%)	366 (92%)	92.2%	
Weekly	472 (43%)	49 (10%)	423 (90%)	89.6%	
Uninformed	8 (1%)	1	7	87.5%	
**Health insurance**					0.23
No	595 (55%)	62 (10%)	533 (90%)	89.6%	
Yes	483 (45%)	40 (8.3%)	443 (92%)	91.7%	
Uninformed	9 (1%)	1	8	88.9%	
**Currently working**					0.26
No	528 (49%)	43 (8.1%)	485 (92%)	91.9%	
On the island	291 (27%)	33 (11%)	258 (89%)	88.7%	
Off the island	160 (15%)	19 (12%)	141 (88%)	88.1%	
Mixed	63 (6%)	4 (6.3%)	59 (94%)	93.7%	
Uninformed	45 (4%)	4	41	91.1%	
**Received government financial assistance in the pandemic**					0.89
No	639 (59%)	57 (8.9%)	582 (91%)	91.1%	
Yes	191 (18%)	16 (8.4%)	175 (92%)	91.6%	
Uninformed	257 (24%)	30	227	88.3%	
**Study location**					< 0.001
On the island	22 (2%)	9 (41%)	13 (59%)	59.1%	
Off the island	36 (3%)	6 (17%)	30 (83%)	83.3%	
Not student	828 (76%)	74 (8.9%)	754 (91%)	91.1%	
Uninformed	201 (18%)	14	187	93.0%	
**Study modality in the pandemic**					< 0.001
Online	41 (4%)	7 (17%)	34 (83%)	82.9%	
In person	17 (2%)	8 (47%)	9 (53%)	52.9%	
Not student	828 (76%)	74 (8.9%)	754 (91%)	91.1%	
Uninformed	201 (18%)	14	187	93.0%	
**Previous history of COVID-19 infection**					0.003
No history	825 (76%)	90 (11%)	735 (89%)	89.1%	
Clinical episode, unconfirmed	129 (12%)	11 (8.5%)	118 (91%)	91.5%	
Lab confirmed clinical episode	129 (12%)	2 (1.6%)	127 (98%)	98.4%	
Uninformed	4 (0%)	0	4	100.0%	
**Household contact of COVID-19 case**					0.32
No	762 (70%)	76 (10.0%)	686 (90%)	90.0%	
Yes	312 (29%)	25 (8.0%)	287 (92%)	92.0%	
Uninformed	13 (1%)	2	11	84.6%	
**Number of COVID-19 cases in the household**					0.51
0	762 (70%)	76 (10.0%)	686 (90%)	90.0%	
1	189 (17%)	15 (7.9%)	174 (92%)	92.1%	
2	70 (6%)	8 (11%)	62 (89%)	88.6%	
3+	47 (4%)	2 (4.3%)	45 (96%)	95.7%	
Uninformed	19 (2%)	2	17	89.5%	
**Number of CONFIRMED COVID-19 cases in the household**					0.83
0	82 (8%)	7 (8.5%)	75 (91%)	91.5%	
1	136 (13%)	11 (8.1%)	125 (92%)	91.9%	
2+	77 (7%)	6 (7.8%)	71 (92%)	92.2%	
Not applicable (no one was suspected)	762 (70%)	76 (10.0%)	686 (90%)	90.0%	
Uninformed	30 (3%)	3	27	90.0%	
**Number of people living in the house (besides the participant)**					0.40
0	133 (12%)	11 (8.3%)	122 (92%)	91.7%	
1	328 (30%)	24 (7.3%)	304 (93%)	92.7%	
2	260 (24%)	29 (11%)	231 (89%)	88.8%	
3	170 (16%)	15 (8.8%)	155 (91%)	91.2%	
4+	179 (16%)	21 (12%)	158 (88%)	88.3%	
Uninformed	17 (2%)	3	14	82.4%	
**SARS-CoV-2 IgG test type**					< 0.001
CMIA anti-S	1,074 (99%)	93 (8.7%)	981 (91%)	91.3%	
POC rapid test	13 (1%)	10 (77%)	3 (23%)	23.1%	
**Vaccination status**					< 0.001
AstraZeneca (one dose >7)	462 (43%)	78 (17%)	384 (83%)	83.1%	
Pfizer (one dose >7)	56 (5%)	3 (5.4%)	53 (95%)	94.6%	
AstraZeneca (two doses >7)	109 (10%)	3 (2.8%)	106 (97%)	97.2%	
CoronaVac (two doses >7)	460 (42%)	19 (4.1%)	441 (96%)	95.9%	
Days since last vaccination**	49 (37; 57)	38 (10; 59)	49 (38; 57)		< 0.001

^*^Pearson's Chi-squared test. Fisher's exact test; ^**^Median (IQR).

**Table 2:** Demographic characteristics of the unvaccinated population (*n* =1832).

		**SARS-CoV-IgG**			
**Variable**	**Total (%)**	**Negative *n* (%)**	**Positive *n* (%)**	**Adjusted Seropositivity (%)**	*P*-value*
**Age group (years)**					< 0.001
0–11	292 (16%)	219 (75%)	73 (25%)	25.0%	
12–17	326 (18%)	268 (82%)	58 (18%)	17.8%	
18–59	1,211 (66%)	779 (64%)	432 (36%)	35.7%	
60+	3 (0%)	2 (67%)	1 (33%)	33.3%	
**Sex**					0.88
Female	908 (50%)	627 (69%)	281 (31%)	30.9%	
Male	924 (50%)	641 (69%)	283 (31%)	30.6%	
**Arterial hypertension**					0.004
No	1,755 (96%)	1,226 (70%)	529 (30%)	30.1%	
Yes	77 (4%)	42 (55%)	35 (45%)	45.5%	
**Diabetes mellitus**					0.59
No	1,816 (99%)	1,258 (69%)	558 (31%)	30.7%	
Yes	16 (1%)	10 (62%)	6 (38%)	37.5%	
**Other comorbidities**					0.001
No	1,628 (89%)	1,107 (68%)	521 (32%)	32.0%	
Yes	204 (11%)	161 (79%)	43 (21%)	21.1%	
**Frequency of leaving the island**					< 0.001
Does not usually go out	356 (19%)	267 (75%)	89 (25%)	25.0%	
1–2 times a month	630 (34%)	455 (72%)	175 (28%)	27.8%	
Weekly	814 (44%)	521 (64%)	293 (36%)	36.0%	
Uninformed	32 (2%)	25	7	21.9%	
**Health insurance**					0.07
No	1,162 (63%)	788 (68%)	374 (32%)	32.2%	
Yes	652 (36%)	469 (72%)	183 (28%)	28.1%	
Uninformed	18 (1%)	11	7	38.9%	
**Currently working**					< 0.001
No	368 (20%)	253 (69%)	115 (31%)	31.3%	
On the island	418 (23%)	261 (62%)	157 (38%)	37.6%	
Off the island	287 (16%)	170 (59%)	117 (41%)	40.8%	
Mixed	100 (5%)	70 (70%)	30 (30%)	30.0%	
Not applicable (< 18 years)	618 (34%)	487 (79%)	131 (21%)	21.2%	
Uninformed	41 (2%)	27	14	34.1%	
**Received government financial assistance in the pandemic**					< 0.001
No	550 (30%)	342 (62%)	208 (38%)	37.8%	
Yes	443 (24%)	290 (65%)	153 (35%)	34.5%	
Not applicable (< 18 years)	618 (34%)	487 (79%)	131 (21%)	21.2%	
Uninformed	221 (12%)	149	72	32.6%	
**Study location**					< 0.001
On the island	471 (26%)	362 (77%)	109 (23%)	23.1%	
Off the island	377 (21%)	276 (73%)	101 (27%)	26.8%	
Not student	754 (41%)	481 (64%)	273 (36%)	36.2%	
Uninformed	230 (13%)	149	81	35.2%	
**Study modality in the pandemic**					< 0.001
Online	517 (28%)	407 (79%)	110 (21%)	21.3%	
In person	291 (16%)	201 (69%)	90 (31%)	30.9%	
Not student	754 (41%)	481 (64%)	273 (36%)	36.2%	
Uninformed	270 (15%)	179	91	33.7%	
**Previous history of COVID-19 infection**					< 0.001
No history	1,317 (72%)	1,018 (77%)	299 (23%)	22.7%	
Clinical episode, unconfirmed	289 (16%)	168 (58%)	121 (42%)	41.9%	
Lab confirmed clinical episode	210 (11%)	70 (33%)	140 (67%)	66.7%	
Uninformed	16 (1%)	12	4	25.0%	
**Household contact of COVID-19 case**					< 0.001
No	1,064 (58%)	813 (76%)	251 (24%)	23.6%	
Yes	743 (41%)	439 (59%)	304 (41%)	40.9%	
Uninformed	25 (1%)	16	9	36.0%	
**Number of COVID-19 cases in the household**					< 0.001
0	1,064 (58%)	813 (76%)	251 (24%)	23.6%	
1	387 (21%)	246 (64%)	141 (36%)	36.4%	
2	210 (11%)	119 (57%)	91 (43%)	43.3%	
3+	131 (7%)	64 (49%)	67 (51%)	51.1%	
Uninformed	40 (2%)	26	14	35.0%	
**Number of CONFIRMED COVID-19 cases in the household**					< 0.001
0	250 (14%)	174 (70%)	76 (30%)	30.4%	
1	280 (15%)	160 (57%)	120 (43%)	42.9%	
2+	170 (9%)	78 (46%)	92 (54%)	54.1%	
Not applicable (no one was suspected)	1,064 (58%)	813 (76%)	251 (24%)	23.6%	
Uninformed	68 (4%)	43	25	36.8%	
**Number of people living in the house (besides the participant)**					0.63
0	63 (3%)	45 (71%)	18 (29%)	28.6%	
1	195 (11%)	144 (74%)	51 (26%)	26.2%	
2	435 (24%)	301 (69%)	134 (31%)	30.8%	
3	509 (28%)	353 (69%)	156 (31%)	30.6%	
4+	598 (33%)	406 (68%)	192 (32%)	32.1%	
Uninformed	32 (2%)	19	13	40.6%	
**SARS-CoV-2 IgG test type**					< 0.001
CMIA anti-S	1,179 (64%)	754 (64%)	425 (36%)	36.0%	
POC rapid test	653 (36%)	514 (79%)	139 (21%)	21.3%	

^*^Pearson's Chi-squared test; Fisher's exact test.

### Risk factors to SARS-CoV-2 seropositivity in non-vaccinated individuals

Epidemiological variables associated with the presence of infection-induced antibodies were analyzed using the unvaccinated group with complete information available. Results of the multivariate logistic regression are shown on Table 3, with 1,783 participants included. This group is younger than the whole population sampled for the study, reflecting the prioritization of the elderly in the vaccine rollout, but we still have representation from all age groups. Many variables were associated with seropositivity on a univariate analysis, but on a multivariate model only three remained associated. History of previous COVID-19 infection [OR = 4.7; (95% CI: 3.25, 7.0)], and being a household contact of a case [OR = 1.9; (95% CI: 1.5, 2.5)] were both associated with the presence of SARS-CoV-2 antibodies. For the students in our sample, in-person learning was also associated with seropositivity [OR = 2.0; (95% CI: 1.4, 3.0)].

**Table 3:** Logistic regression of risk factors to SARS-CoV-2 seropositivity in unvaccinated residents (*n* = 1,783).

			**Univariate**		**Multivariate**	
**Variable**	**Total (%)**	**SARS-CoV-IgG + (%)**	**OR (CI)**	***P*-value**	**OR (CI)**	***P*-value**
**Age**						
0–11	292 (16%)	73 (25%)	—		—	
12–17	326 (18%)	58 (18%)	0.65 (0.44, 0.96)	0.029	0.62 (0.39, 0.99)	0.047
18–49	1110 (62%)	394 (35%)	1.24 (0.86, 1.80)	0.2	1.53 (0.94, 2.51)	0.086
50+	55 (3%)	22 (40%)	2 (1.09, 3.63)	0.024	2.35 (0.97, 5.62)	0.055
**Arterial hypertension**						
No	1717 (96%)	517 (30%)	—		—	
Yes	66 (4%)	30 (45%)	1.93 (1.17, 3.17)	0.009	1.41 (0.74, 2.65)	0.3
**Other comorbidities**						
No	1585 (89%)	504 (32%)	—		—	
Yes	198 (11%)	43 (22%)	0.6 (0.41, 0.84)	0.004	0.67 (0.43, 1.02)	0.065
**Frequency of leaving the island**						
Does not usually go out	348 (20%)	85 (24%)	—		—	
1–2 times a month	615 (34%)	174 (28%)	1.22 (0.91, 1.65)	0.2	1.03 (0.72, 1.48)	0.9
Weekly	789 (44%)	282 (36%)	1.72 (1.30, 2.30)	< 0.001	1.1 (0.75, 1.62)	0.6
Uninformed	31 (2%)	6				
**Currently working**						
No	350 (20%)	113 (32%)	—		—	
On the island	399 (22%)	149 (37%)	1.25 (0.92, 1.69)	0.15	1.09 (0.75, 1.60)	0.7
Off the island	280 (16%)	114 (41%)	1.44 (1.04, 2.00)	0.029	1.07 (0.70, 1.63)	0.8
Mixed	97 (5%)	28 (29%)	0.85 (0.51, 1.38)	0.5	0.78 (0.41, 1.45)	0.4
Not applicable (< 18 years)	618 (35%)	131		< 0.001		
Uninformed	39 (2%)	12				
**Currently studying**						
No	723 (41%)	263 (36%)	—			
Yes	851 (48%)	213 (25%)	0.58 (0.47, 0.72)	< 0.001		
Uninformed	209 (12%)	71				
**Study location**						
On the island	467 (26%)	108 (23%)	—		—	
Off the island	375 (21%)	101 (27%)	1.23 (0.90, 1.68)	0.2	0.92 (0.60, 1.41)	0.7
Not student	723 (41%)	263 (36%)	1.9 (1.46, 2.48)	< 0.001	1.3 (0.84, 2.02)	0.2
Uninformed	218 (12%)	75				
**Study modality in the pandemic**						
Online	514 (29%)	110 (21%)	—		—	
In person	289 (16%)	89 (31%)	1.63 (1.18, 2.27)	0.003	2.01 (1.37, 2.94)	< 0.001
Not student	723 (41%)	263				
Uninformed	257 (14%)	85				
**Previous history of COVID-19 infection**						
No history	1287 (72%)	293 (23%)	—		—	
Clinical episode, unconfirmed	280 (16%)	116 (41%)	2.4 (1.83, 3.14)	< 0.001	1.88 (1.37, 2.59)	< 0.001
Lab confirmed clinical episode	202 (11%)	135 (67%)	6.84 (4.98, 9.46)	< 0.001	4.74 (3.25, 6.97)	< 0.001
Uninformed	14 (1%)	3				
**Household contact of COVID-19 case**						
No	1035 (58%)	244 (24%)	—		—	
Yes	724 (41%)	295 (41%)	2.23 (1.81, 2.74)	< 0.001	1.93 (1.48, 2.51)	< 0.001
Uninformed	24 (1%)	8				
**Number of COVID-19 cases in the household**						
0	1035 (58%)	244 (24%)	—			
1	376 (21%)	137 (36%)	1.86 (1.44, 2.40)	< 0.001		
2	206 (12%)	88 (43%)	2.42 (1.77, 3.30)	< 0.001		
3+	127 (7%)	65 (51%)	3.4 (2.33, 4.96)	< 0.001		
Uninformed	39 (2%)	13				
**Number of CONFIRMED COVID-19 cases in the household**						
0	244 (14%)	74 (30%)	—			
1	272 (15%)	115 (42%)	1.68 (1.17, 2.43)	0.005		
2+	167 (9%)	90 (54%)	2.69 (1.79, 4.05)	< 0.001		
No one was suspected	1035 (58%)	244 (24%)	0.71 (0.52, 0.97)	0.029		
Uninformed	65 (4%)	24				
**SARS-CoV-2 IgG test type**						
CMIA anti-S	1135 (64%)	410 (36%)	—			
POC rapid test	648 (36%)	137 (21%)	0.47 (0.38, 0.59)	< 0.001		

### Antibody titers on the seropositive participants

**Figure 1:**
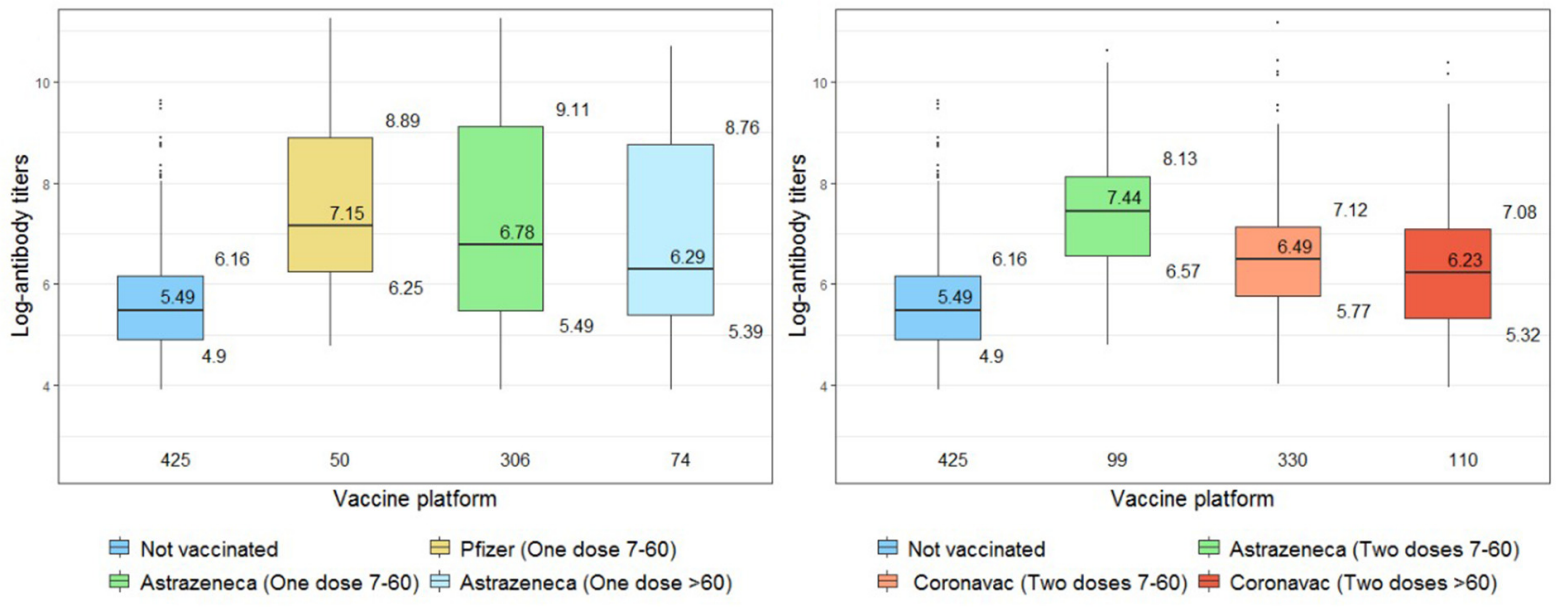
Comparison of log-antibody titers across groups of vaccine exposure (restricted to seropositive individuals).

Comparison of the log-antibody titers on the seropositive participants tested with the quantitative CMIA (*n* = 1,370) is shown in Figure 1 and Table 4. We further stratified the vaccinated participants with one dose of AstraZeneca and two doses of CoronaVac, separating those who have received the last vaccine dose between 7 and 60 days and those who received it more than 60 days before the survey. All those with two doses of AstraZeneca and also those with one dose of Pfizer had < 60 days since the last dose. The group of seropositive unvaccinated participants, with infection-induced antibodies, had lower titers than all the vaccinated groups. Being vaccinated with Pfizer or AstraZeneca [Beta = 2.2; CI = (1.8, 2.6)] determined higher log-antibody titers than those observed with CoronaVac [Beta = 1.2; CI = (0.88, 1.5)]. Since the regression coefficient is the difference in logarithm between comparison groups, it corresponds to the ratio of antibody titers. For instance, those vaccinated with one dose of the AstraZeneca vaccine had antibody titers 10^2.2^ (=158) times as high as unvaccinated individuals. For CoronaVac the ratio was 15.8. The groups that had received the last dose more than 60 days before the survey showed smaller differences in log-antibody titers (lower antibody titer ratios), particularly the CoronaVac vaccines [Beta = 0.93; CI = (0.56, 1.3)].

**Table 4:** Multivariate linear regression analysis of log-antibody titers among seropositive participants with quantitative serological tests available (*n* = 1,370).

**Risk factor**	**Total**	**Beta (CI)**	***P*-value**
**Previous history of COVID-19 infection**			
No history	912	—	
Clinical episode, unconfirmed	213	0.50 (0.28, 0.72)	< 0.001
Lab confirmed clinical episode	245	1.0 (0.82, 1.2)	< 0.001
**Household contact of COVID-19 case**			
No	883	—	
Yes	487	0.35 (0.18, 0.52)	< 0.001
**Vaccination status**			
Unvaccinated	416	—	
Pfizer (one dose, 7–60 days)	50	2.2 (1.8, 2.6)	< 0.001
AstraZeneca (two doses 7–60 days)	97	2.2 (1.8, 2.6)	< 0.001
AstraZeneca (one dose, 7-60 days)	300	1.7 (1.5, 2.0)	< 0.001
AstraZeneca (one dose, >60 days)	72	1.4 (1.0, 1.8)	< 0.001
CoronaVac (two doses, 7–60 days)	327	1.2 (0.88, 1.5)	< 0.001
CoronaVac (two doses >60 days)	108	0.93 (0.56, 1.3)	< 0.001

**Figure 2:**
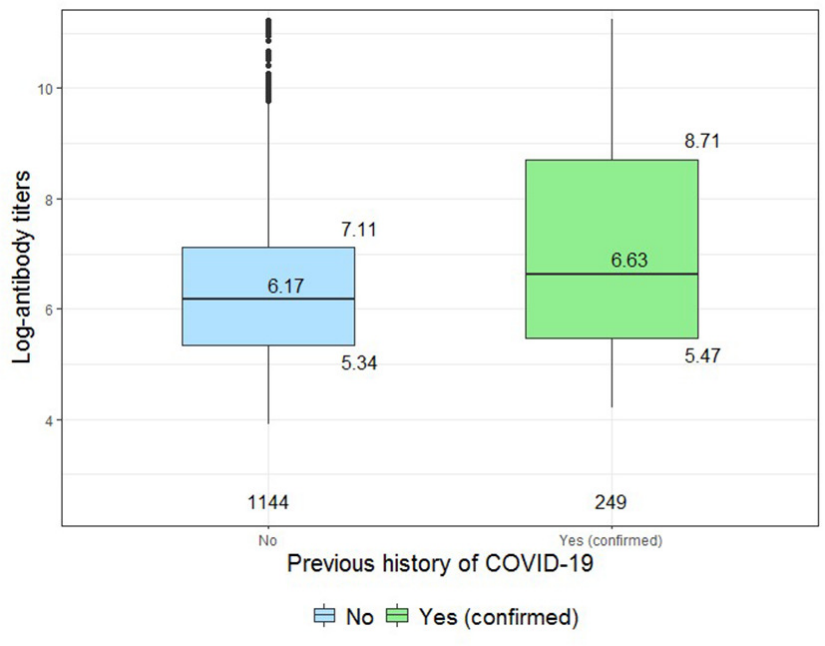
Comparison of log-antibody titers between vaccinated participants with or without history of previous SARS-CoV-2 infection (restricted to vaccinated and seropositive individuals).

History of COVID-19 was independently associated with higher antibody titers on the model (Table 4), and Figure 2 shows how hybrid immunity was superior to vaccine-induced immunity when comparing titers only on vaccinated participants according to previous infection exposure.

## Discussion

In this seroepidemiologic survey we included 83.5% of Paquetá Island's residents, thus achieving a fair representation of the population. Of note, we had a higher participation of children and adolescents than previous studies of seroprevalence (13–15).

Compared to surveys conducted in Brazil around mid-2021 the seroprevalence of antibodies against SARS-CoV-2 in our sample (53.61%) was higher than other studies reported in the South and Southeast regions of Brazil (14, 16, 17), being closer to rates observed at that time in the North region (18, 19) and in indigenous populations (20, 21). With a vaccine coverage of 37% in our study population, the large difference in seroprevalence between vaccinated (90.5%) and unvaccinated (31%) residents confirmed findings from other studies (22) of how vaccination decisively contributes to the population's immunity, even in an area with a high prevalence of infection-induced antibodies after exposure to a pandemic for more than a year.

Surveillance data from the beginning of the pandemic to the date of the survey showed a 40% higher cumulative incidence in the Paquetá Island compared to the rest of the city. This difference could be explained by epidemiological differences in the pattern of contacts among residents of the Island, but also by a lag in the population estimate for the city of Rio de Janeiro, which based on data from the 2010 census, while the Island has data from a census carried out by the IBGE in 2022, as a test for the next national census. Despite the reported high incidence on the Island, this may still be underestimated, considering that it was calculated based on 288 surveillance reported cases in Paquetá in the period, while on our sample (83.5% of the population) 339 residents reported a previous laboratory confirmed COVID-19 infection, and only among the unvaccinated participants (50.7% of the Paquetá population) 564 seropositive residents were identified. This illustrates both the large number of asymptomatic infections and the underreporting of symptomatic cases. Also of note was that 21% of participants who reported lab-confirmed clinical episodes were seronegative, showing that antibody levels from natural infection decreased to levels below the test threshold (assuming errors in lab-confirmation are negligible).

The analysis of the unvaccinated participants allowed us to assess risk factors for natural infection. With higher seropositivy among adults, followed by children and adolescents, age seems to determine the risk of infection even in a community with more restricted mobility such as Paquetá Island. The association of self-reported COVID-19 infection with seropositivity in the regression model illustrates the usefulness of the clinical history for epidemiological analysis even in cases without laboratory confirmation. It is also interesting to note the importance of being a household contact of cases as a risk factor, as already observed in other studies. In contrast, the number of residents in the same household did not present a correlation as was reported in other publications. Our results also demonstrate how online learning was significantly associated with the absence of antibodies in unvaccinated students, suggesting it was a successful strategy to prevent exposure to COVID-19. This may help to guide decision-making when considering social distancing control measures.

COVID-19 vaccines roll-out started in Brazil on January 17, 2021, and by the time of our survey, 5 months later, three different vaccines had been used in Rio de Janeiro: AstraZeneca, Pfizer and CoronaVac, all approved only for people 18 years old and above. Antibody titers are highly correlated with neutralizing antibodies, which have been associated with COVID-19 vaccines efficacy, and our immunogenicity results for the three vaccines were in line with the efficacy observed in randomized clinical trials (23–25). Time since last dose, number of doses, and vaccine brand were all strongly associated with magnitude of serological response. The drop in titers after 60 days of the first dose of AstraZeneca suggests the need for a better definition of interval between doses, as this vaccine was approved in Brazil with a broad 4 to 12-week interval. Differences in the antibody response to the three vaccine brands, particularly the low titers 60 days after two doses of CoronaVac, also suggest that the optimal interval to a booster dose could be adjusted by vaccine type. The differences between infection-induced and vaccine-induced antibody titers were clear for all vaccines, even with incomplete schedules, and hybrid immunity was associated with the highest observed titers.

Limitations to our study were the sample size and unique geography of the area, hindering extrapolation to places with different characteristics. The retrospective data on clinical infections and observational uncontrolled data on the mix of vaccine schedules also limits conclusions on determinants of antibody response, particularly considering the time-dependent kinetics of humoral response.

## Conclusions

Our study highlights the positive impact of vaccination on COVID-19 collective immunity even in a highly affected population, while showing the superiority of the hybrid immunity antibody level. It also shows the difference in antibody titers achieved with different vaccines and how they wane with time, reinforcing how these factors should be considered when estimating effectiveness of a vaccination program at any given time. Further analyses should address the indirect protection of unvaccinated groups. Finally, we provide evidence of the impact of online learning, a non-pharmacological intervention, on the incidence of SARS-CoV-2 infections, which may help guide future public health policies.

## Data availability statement

The raw data supporting the conclusions of this article will be made available by the authors, without undue reservation.

## Ethics statement

The studies involving human participants were reviewed and approved by National Research Ethics Committee - CONEP. Written informed consent to participate in this study was provided by the participants' legal guardian/next of kin.

## Author contributions

JC-N and DS conceptualized the study. JC-N, MV, LB, JM, LP, MC, DS, LC, and GP participated in the design of the study. Conduct of the study and data collection were performed by JC-N, MV, LB, JM, LP, MG, and DS. Drafted the initial manuscript and data analysis were performed by JC-N and IP. All authors revised subsequent drafts, read, and approved the final manuscript for submission.

## Funding

This study was supported by the Oswaldo Cruz Foundation (Fiocruz) and the Rio de Janeiro City Department of Health. This work is part of the Grand Challenges ICODA pilot initiative, delivered by Health Data Research UK and funded by the Bill & Melinda Gates Foundation (INV 017293) and the Minderoo Foundation. This study was also supported by the National Council for Scientific and Technological Development (CNPq), the Coordinating Agency for Advanced Training of Graduate Personnel (CAPES; finance code 001), Carlos Chagas Filho Foundation for Research Support of the State of Rio de Janeiro (FAPERJ), and the Pontifical Catholic University of Rio de Janeiro. OR was funded by a Sara Borrell grant from the Instituto de Salud Carlos III (CD19/00110).

## Conflict of interest

Authors JC-N, MV, LB, JM, MC, DS, MM, MK, RC, LC, FB, and GP are employed by Fiocruz, which manufactures the AstraZeneca vaccine in Brazil.

The remaining authors declare that the research was conducted in the absence of any commercial or financial relationships that could be construed as a potential conflict of interest.

## Publisher's note

All claims expressed in this article are solely those of the authors and do not necessarily represent those of their affiliated organizations, or those of the publisher, the editors and the reviewers. Any product that may be evaluated in this article, or claim that may be made by its manufacturer, is not guaranteed or endorsed by the publisher.

## References

[1] *COVID Live - Coronavirus Statistics - Worldometer*. Available online at: https://www.worldometers.info/coronavirus/ (accessed June 13, 2022).

[2] *IBGE | Cidades@ | Rio de Janeiro | Rio de Janeiro | Panorama*. Available online at: https://cidades.ibge.gov.br/brasil/rj/rio-de-janeiro/panorama (accessed June 13, 2022)

[3] IBGE Divulga Dados Preliminares do Teste do Censo na Ilha de Paquetá | Agência de Notí cias. Agência de Notícias - IBGE. (2021). Available online at: https://agenciadenoticias.ibge.gov.br/agencia-noticias/2012-agencia-de-noticias/noticias/31904-ibge-divulga-dados-preliminares-do-teste-do-censo-na-ilha-de-paqueta (accessed June 13, 2022).

[4] *e-SUS Notifica*. Available online at: https://notifica.saude.gov.br/login (accessed June 28, 2022).

[5] *SIVEP - Gripe - Notificações de S*í*ndromes Respiratórias Agudas Graves (SRAG) - Rio de Janeiro*. Available online at: http://sistemas.saude.rj.gov.br/tabnetbd/dhx.exe?sivep_gripe/sivep_gripe.def (accessed June 13, 2022).

[6] SI-PNI. Available online at: https://si-pni.saude.gov.br/#/login (accessed June 28, 2022).

[7] Jung K Shin S Nam M Hong YJ Roh EY Park KU. Performance evaluation of three automated quantitative immunoassays and their correlation with a surrogate virus neutralization test in coronavirus disease 19 patients and pre-pandemic controls. J Clin Lab Anal. (2021) 35:e23921. 10.1002/jcla.23921 34369009 PMC8418513

[8] FDA. EUA Authorized Serology Test Performance. US Food and Drug Administration. (2021). Available online at: https://www.fda.gov/medical-devices/coronavirus-disease-2019-covid-19-emergency-use-authorizations-medical-devices/eua-authorized-serology-test-performance (accessed April 5, 2022).

[9] Azure Biotech Inc. COVID-19 IgG/IgM Rapid Test Device Package Insert. (2022). Available online at: https://www.fda.gov/media/139792/download (accessed June 7, 2022).

[10] Department of health human services USA. Serology Test Evaluation Report for “FaStep Rapid Diagnostic Test Coronavirus Disease 2019/ (COVID-2019) IgG/IgM Rapid Test”. (2020). Available online at: https://www.accessdata.fda.gov/cdrh_docs/presentations/maf/maf3289-a001.pdf (accessed April 4, 2022).

[11] Kohmer N Westhaus S Rühl C Ciesek S Rabenau HF. Clinical performance of different SARS-CoV-2 IgG antibody tests. J Med Virol. (2020) 10:1002. 10.1002/jmv.26145 32510168 PMC7300776

[12] Sempos CT Tian L. Adjusting coronavirus prevalence estimates for laboratory test kit error. Am J Epidemiol. (2020) 190:109–15. 10.1101/2020.05.11.20098202 32803245 PMC7454308

[13] Arora RK Joseph A Wyk JV Rocco S Atmaja A May E. SeroTracker: a global SARS-CoV-2 seroprevalence dashboard. Lancet Infect Dis. (2021) 21:e75–6. 10.1016/S1473-3099(20)30631-9 32763195 PMC7402646

[14] Barros AJD Victora CG Menezes AMB Horta BL Barros FC Hartwig FP. Population-level seropositivity trend for SARS-CoV-2 in Rio Grande do Sul, Brazil. Rev Saúde Pública. (2021) 55:78. 10.11606/s1518-8787.2021055004075 34816981 PMC8577540

[15] Miraglia JL Nascimento Monteiro C Giannecchini Romagnolo A Xavier Gomes R Pitangueiras Mangueira C Aparecida Rosseto-Welter E. A seroprevalence survey of anti-SARS-CoV-2 antibodies among individuals 18 years of age or older living in a vulnerable region of the city of São Paulo, Brazil. PLoS ONE. (2021) 16:e0255412. 10.1371/journal.pone.0255412 34324603 PMC8320930

[16] Huergo LF Paula NM Gonçalves ACA Kluge CHS Marins PHSA Camargo HSC. SARS-CoV-2 seroconversion in response to infection and vaccination: a time series local study in Brazil. Microbiology Spectrum [Internet]. (2022) 10:e01026–22. 10.1101/2022.03.10.22271805 35770982 PMC9430992

[17] Albuquerque JOM de Kamioka GA Madalosso G Costa SA Ferreira PB Pino FA. Prevalence evolution of SARS-CoV-2 infection in the city of São Paulo, 2020–2021. Rev Saúde Pública. (2021) 55:62–62. 10.11606/s1518-8787.2021055003970 34706038 PMC8522737

[18] de Lima ACR Lopes FT de Sousa RS Gomes JLC Pereira KAS de Lima CNC. Avaliação clínico-epidemiológica da infecção pelo SARS-CoV-2 em moradores do município de maracanã, no estado do pará. Braz J Infect Dis. (2022) 26:102011. 10.1016/j.bjid.2021.102011

[19] Nicolete VC Rodrigues PT Fernandes ARJ Corder RM Tonini J Buss LF. Epidemiology of COVID-19 after Emergence of SARS-CoV-2 Gamma Variant, Brazilian Amazon, 2020–2021 - Volume 28, Number 3—March 2022 - Emerging Infectious Diseases journal - CDC. Available online at: https://wwwnc.cdc.gov/eid/article/28/3/21-1993_article (accessed May 26, 2022). 10.3201/eid2803.211993 PMC8888227 34963505

[20] Pereira KAS Brito WR dos S Lopes FT de Lima ACR Lima CNC Souza I de P. Prevalência do coronavírus 2 da síndrome respiratória aguda grave (SARS-CoV-2) em comunidades quilombolas do munícipio de cametá, pará Braz. J Infect Dis. (2022) 26:102060. 10.1016/j.bjid.2021.102060

[21] Lima CNC Abreu IN Rodrigues EPS Freitas V de O Botelho BJS Souza SL. Anti-SARS-CoV-2 antibodies among indigenous populations of the Brazilian Amazon: a cross-sectional study. BMJ Open. (2022) 12:e054271. 10.1136/bmjopen-2021-054271 35131827 PMC8822535

[22] Whitaker HJ Elgohari S Rowe C Otter AD Brooks T Linley E. Impact of COVID-19 vaccination program on seroprevalence in blood donors in England, 2021. J Infect. (2021) 83:237–79. 10.1016/j.jinf.2021.04.037 33989631 PMC8110696

[23] Palacios R Batista AP Albuquerque CSN Patiño EG Santos J. do P, Tilli Reis Pessoa Conde M, et al. Efficacy and safety of a COVID-19 inactivated vaccine in healthcare professionals in Brazil: The PROFISCOV study. SSRN J. (2021). 10.2139/ssrn.3822780

[24] Polack FP Thomas SJ Kitchin N Absalon J Gurtman A Lockhart S. Safety and efficacy of the BNT162b2 mRNA Covid-19 vaccine. N Engl J Med. (2020) 383:2603–15. 10.1056/NEJMoa2034577 33301246 PMC7745181

[25] Voysey M Clemens SAC Madhi SA Weckx LY Folegatti PM Aley PK. Safety and efficacy of the ChAdOx1 nCoV-19 vaccine (AZD1222) against SARS-CoV-2: an interim analysis of four randomised controlled trials in Brazil, South Africa, and the UK. Lancet Lond Engl. (2021) 397:99–111. 10.1016/S0140-6736(20)32661-1 33306989 PMC7723445

