# Supplementary material for: Seroepidemiology of SARS-CoV-2 on a partially vaccinated island in Brazil: Determinants of infection and vaccine response
Source: Front Public Health. 2022 Nov 14;10:1017337. doi: 10.3389/fpubh.2022.1017337 (PMC9706255; doi:10.3389/fpubh.2022.1017337)
Supplement: Supplementary file 1 [file Data_Sheet_1.docx]

Supplementary Material

# Supplementary Data

**Supplementary Figure 1.** COVID-19 cumulative incidence rate and vaccination coverage on Rio de Janeiro City (RJC) and on Paquetá Island, March 2020 – June 2021.

**Supplementary Figure 2.** SARS-CoV-2 variants distribution by month of isolation on sentinel genomic surveillance in Rio de Janeiro City, March 2020 – June 2021.


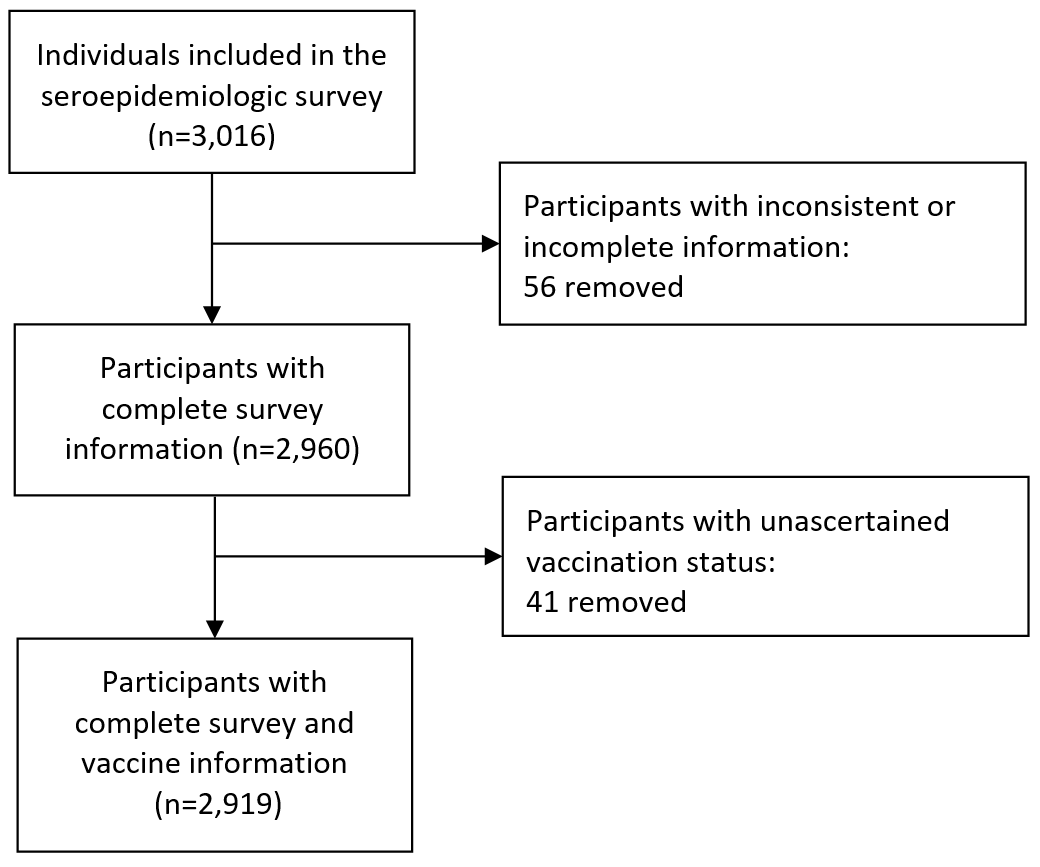


**Supplementary Figure 3.** Flowchart for study population inclusion
